# Antibiotic treatment failure when consulting patients with respiratory tract infections in general practice. A qualitative study to explore Danish general practitioners’ perspectives

**DOI:** 10.1080/13814788.2017.1305105

**Published:** 2017-04-10

**Authors:** Margrethe Bordado Sköld, Rune Aabenhus, Ann Dorrit Guassora, Marjukka Mäkelä

**Affiliations:** ^a^ Center for Education and Research in General Practice, Department of Public Health, Faculty of Health Sciences, University of CopenhagenCopenhagenDenmark; ^b^ Finnish Office for Health Technology Assessment (FINOHTA), THL (National Institute for Health and Welfare)HelsinkiFinland

**Keywords:** Antibiotic treatment failure, doctor–patient relationship, general practice, acute respiratory tract infection, qualitative study

## Abstract

**Background:** Prescribing antibiotics for acute respiratory tract infections (RTIs) is common in primary healthcare although most of these infections are of viral origin and antibiotics may not be helpful. Some of these prescriptions will not be associated with a quick recovery, and might be regarded as cases of antibiotic treatment failure (ATF).

**Objectives:** We studied antibiotic treatment failure in patients with acute RTIs from a general practitioner (GP) perspective, aiming to explore (i) GPs’ views of ATF in primary care; (ii) how ATF influences the doctor-patient relationship; and (iii) GPs’ understanding of patients’ views of ATF.

**Methods:** Qualitative study based on semi-structured, recorded interviews of 18 GPs between August and October 2012. The interviews started with discussion of a unique case of acute RTI involving ATF, followed by a more general reflection of the topic. Interviews were analysed using qualitative content analysis.

**Results:** In patients with acute RTIs, GPs proposed and agreed to a medical definition of antibiotic treatment failure but believed patients’ views to differ significantly from this medical definition. GPs thought ATF affected their daily work only marginally. GPs used many communicative tools to maintain trust with patients in cases of ATF, but they did not consider such incidents to affect the doctor-patient relationship adversely.

**Conclusion:** These findings suggest a possible communication gap between doctors and patients, partly due to a narrow medical definition of ATF. Studies describing patients’ views are still missing.

General practitioners’ experiences and views on antibiotic treatment failure in acute respiratory infections or its effects on the doctor–patient relationship have not been studied previously.

KEY MESSAGES

- GPs do not consider antibiotic treatment failure to be a major problem in their work.
- Communication is a key tool in maintaining a good doctor–patient relationship in case of antibiotic treatment failure.
- GPs believe patients’ views of antibiotic treatment failure might differ from doctors’ views.

## Introduction

Acute respiratory tract infections (RTIs) are among the most frequent reasons for consultations in primary healthcare [1]. In Denmark, as in many Northern European countries, antibiotics are generally prescribed with caution and only after thorough consideration. Several campaigns have educated both general practitioners (GPs) and patients to avoid unnecessary antibiotic prescriptions, especially broad-spectrum agents, as we know that 90% of antibiotics in Denmark are prescribed in primary healthcare [2,3]. Most (80%) of acute RTIs in general practice are caused by viral infections where antibiotics are not needed or of little benefit [4–6]. Discriminating between viral and bacterial aetiologies is virtually impossible [7], so doctors often err on the side of caution and prescribe antibiotics. This happens in half of the RTI cases in primary care in Denmark, and in up to 70% in some European countries [8]. High prescribing rates are worrisome as excessive antibiotic use contributes to increasing levels of antibiotic resistance [9], which in turn is associated with considerable morbidity and mortality, especially from multi-resistant bacteria [10].

When no clinical improvement is apparent despite compliance with a course of antibiotics, patients and GPs may perceive this as a case of antibiotic treatment failure (ATF). Cases of ATF often cause additional doctor–patient contacts where both patient and GP may reconsider treatment decisions. This could affect the doctor–patient relationship negatively. Good communication can reduce antibiotic prescription in general practice [11]. Thus, we found it interesting to investigate ATF and its impact on the doctor–patient relationship.

There is, however, no consensus on a definition of ATF in primary care [12], probably due to inherent problems of defining the aetiology of infections and the fact that many bacterial infections are not serious but self-limiting so the benefit from antibiotics is difficult to assess. Currie et al., using a pragmatic definition of ATF cases (first line antibiotic treatment replaced by a new antibiotic within 30 days), found an antibiotic failure rate of 15.4% [13].

GPs’ understanding of ATF in their daily work has apparently not been studied earlier.

The objectives of this study were to describe GPs’ definitions of ATF and the challenges it poses to doctor–patient relationships, and to elicit GPs’ assumptions of their patients’ understanding of ATF.

## Methods

All interviews were conducted in Copenhagen, Denmark, from August to October 2012. We chose a qualitative study approach, as these methods are especially useful for studying experiences and attitudes [14]. Ethical approval for interview studies of health professionals is not required in Denmark. According to the legislation on ethical evaluation of medical research projects in Denmark (Law 593 of 14 June 2011), questionnaire studies are evaluated by ethics committees only if they also involve samples of biological materials.

### Participants

Participants were recruited using operational construct sampling [15], i.e. a focused sampling based on our theories of interests. To get a broad range of views on the topic, we aimed at variation in participant age, gender and clinical experience. For practical reasons, only clinics located in the Copenhagen area and Zealand region were targeted. Email and/or letter invitations were sent out to a broad range of clinics, no reminders were sent and no compensation for participation was given. Interested GPs received both written and oral information about the study. No GPs refused to participate at this point. Our sample size determination (*n* = 15–20) was based on the sparse existing theoretical background on ATF, lack of previous experience of interviewer and a rather narrow study aim [16]. This estimation was retained after a preliminary review of data from the first three interviews (MBS, ADG). Since no new information appeared in the last three interviews, we closed the data collection, ending on 18 participants.

### Interviews

Data were collected through semi-structured qualitative interviews [15,17]. One author (MBS) conducted the interviews face-to-face except for one that was done by telephone (GP 3). The interviewer was a medical student at the time of data collection and had no previous research experience. She had caught interest in the subject from the patients’ perspective, assuming ATF would have a large impact on the doctor–patient relationship. The interviewer’s role was dynamic and evolved during the course of interviews. The semi-structured interview guide (Table 1) ensured consistency in data collection. The guide had open-ended questions to ensure that topics of interest were systematically explored while allowing the interviewees to answer in their own words and present their personal views [15,17].

**Table 1.** Interview guide.

Questions based on if the GP remembered a specific, relevant case	Questions on the topic in general (both in relation to the GPs’ own definition of ATF and when consulting patients with acute RTIs)
Do you remember a specific case of antibiotic treatment failure? If so: Please describe case in detail, here including your understanding of antibiotic treatment failure. Did you expect a possible treatment failure or did the treatment failure come unexpectedly? Any following complication or problems. The patient’s reaction. Did your image of yourself as a doctor change? Was the doctor–patient relationship (trust) affected? Additional comments on the case.	Do you consider antibiotic treatment failure a general problem in general practice (your own practice/generally)? Any common features of occurring cases. Acute respiratory tract infection vs other infections —does it differ? Which factors determine the need of an antibiotic prescription? In cases of doubt—prescribe or withhold? Why do you believe this topic (antibiotic treatment failure) is not more examined or discussed? Do you find that there is a need for increased focus, debate or research? Additional comments.

One interview included two GPs at the same time (GPs 5 and 6); each of these was analysed individually from the transcript as far as possible. Interviews took place in the GP’s own clinic or similar available office facilities. We told GPs that our purpose was not to audit practice but to understand their experiences, actions and reasoning concerning antibiotic treatment failure in acute respiratory tract infections (ICD-10 codes DJ00–DJ22).

In the first part of the interview, GPs were asked to recall a specific case of ATF in an acute RTI patient and describe it in detail. The second part addressed GPs’ thoughts and views of ATF in general, in relation to their own definition, and when seeing patients with acute RTIs. The length of the interviews varied between 15 and 30 min. All interviews were digitally recorded and the interviewer transcribed them verbatim the same or the following day.

### Analysis

All transcriptions were thoroughly read and reread to obtain a sense of whole before starting the coding process to identify relevant data. The analysis of the interviews was based on qualitative content analysis [18], a method using inductive category formation, with a focus on the manifest content. Three researchers independently (MBS, RA, ADG) coded the first three interviews and provisional categories were developed. An example of a meaning unit turned into codes is in Box 1.

**Box 1.** Example of the process of analysis, turning meaning unit into codes.

MEANING UNIT		CODES
‘I don’t know how many treatment failures there are. I have my own clinical experience, and I don’t encounter many treatment failures there. I encounter perhaps more uncertain diagnoses, or that the treatment was initiated on a loose indication. And then I find that it is perhaps more diagnostic failure than treatment failure right?’	→	I. Overall treatment failure is not a great concern/is unimportant.
	→	II. Treatment failure is hard to define and ‘get a hold of’.

The main difference in the method used in this study and the one suggested by Graneheim and Lundman is the absence of condensed meaning unit, and instead, the creation of codes directly from the original text [18]. Considering the manageable amount of gathered data in this study (18 brief interviews), we regarded it acceptable to code directly from the text.

The process of analysis was dynamic, with codes and categories remodelled throughout the analysis of interviews. We kept focus on the content as a whole throughout the process. Analysis was primarily performed by MBS together with ADG who mentored the process and control coded some of the interviews for comparison. Any differences between the researchers were discussed and reflected upon. Between all authors, the categories were continuously revised and possible changes conferred, until agreement was reached. An example of the relationship between codes, subcategories, and category is shown in Table 2.

**Table 2.** Example of theme, category, subcategories and some belonging codes.

Theme	Antibiotic treatment			
Category	Factors of influence			
Sub-categories	Scientific influence	Influence due to patient characteristics	Influence due to patient demand	Influence due to other factors
Codes	Guidelines followed as far as possible Advice from colleagues/specialists The issue of bacterial resistance is essential The overall clinical impression of the patient is essential	Expectations of low patient compliance The patient’s general health condition (age, immunologically challenged, chronic illness)	Requests for children from their parents Requests due to weekends, holidays and other events Habits from patient’s former GP Expectations of simple and effective treatment	Time pressure Seasonal variation/epidemics Price Being tired when working/lacking energy to argue Experience changing GPs’ perspective gradually

During interviews, it would happen that GPs first made a statement and later said something opposing this. This would often relate to acting differently in similar situations; instead of representing a contradiction, it illustrates the diversity in GPs’ work and uniqueness of patient encounters, which many GPs pointed out. These dilemmas were included in the analysis.

## Results

Altogether 18 GPs were interviewed, all from private health clinics, which is the standard way of running a GP clinic in Denmark. Fourteen worked in clinics in the urban Copenhagen area and four in more rural clinics on Zealand. Eleven GPs were involved in research. Participant background is presented in Table 3.

**Table 3.** Baseline characteristics of participants.

GP	Gender	Years as GP
1	Male	0^a^
2	Male	0^a^
3	Female	1
4	Male	0^a^
5	Female	1
6	Female	21
7	Male	35
8	Male	30
9	Male	32
10	Female	0^a^
11	Female	12
12	Male	12
13	Male	20
14	Female	23
15	Male	9
16	Female	24
17	Male	20
18	Female	20

^a^ In specialist training.

### Antibiotic treatment failure—definition and influence on doctor–patient relationship

GPs did overall agree upon a medical interpretation of ATF: a bacterial infection where the prescribed antibiotic is not effective, due to incorrect type or inadequate dose of antibiotic or to antimicrobial resistance. Most GPs argued that in practice it is almost impossible to verify if treatment failure actually occurred or not and they generally considered ATF to be unavoidable in primary care. According to this medical definition, many GPs argued that to avoid ATF the only alternative would be prescribing broad-spectrum antibiotics in first RTI consultations. Considering the risk of antibiotic resistance most did not approve of this option and preferred to start out with narrow-spectrum agents, running the risk of ATF instead. A younger GP illustrated the inevitability of ATF in the following statement:

It’s one of those dogmas you are dealing with, like it’s fundamental. There is lots of uncertainty in a physician’s decision, and you do some sort of probability evaluation in your diagnosis, and then you treat from that. However, sometimes there will be a change in treatment. I do not think there is anything strange in that. Surely you cannot hit right every time. (GP 1)

In cases where changes in treatment would be needed, the GPs’ impression was that patients accepted the change, especially if they were informed of this possibility beforehand. GPs therefore felt that trust between patient and doctor was rarely affected. A young doctor claimed:

And in fact, I find that they are quite understanding towards it. That now, we will start out with this, regular penicillin. However, something else, another type, could be required. I have not had anyone saying, “but then I would just like the other one to begin with.” So when you prepare them for it, I find that it runs pretty smoothly. (GP 4)

Some GPs, however, had experienced cases of ATF affecting the doctor–patient relationship negatively, with patients becoming impatient and angry. Contrastingly, others told of episodes where the relationship, due to increased number of consultations, testing and monitoring, evolved in a positive direction as the patient felt well cared for even after an initial disappointment.

GPs did not consider patients’ possible frustration and dissatisfaction due to ATF very important. Most cases of ‘treatment failure’ involving frustration from the patients were actually not caused by ATF but impatience and ignorance about the course of the illness, the GPs reflected.

### GPs’ management strategies

GPs mentioned several issues in RTI consultations that consumed time and energy, could affect the doctors’ approach, and influence treatment choices.

#### Communication issues

The GPs saw consultations for acute RTIs as distinctive since patients and doctors often had different views on management of RTIs. Many GPs stated that patients were generally not aware of the variable aetiology of infections, and thus considered antibiotics equally effective and needed in both viral and bacterial infections. Generally, patients expected medication that would get their children or themselves quickly back to school or work. GPs claimed it took time to explain that antibiotics often were unnecessary and that symptoms would last equally long with or without antibiotics.

When given a thorough explanation for not receiving a prescription, most patients would agree. A young, newly educated GP stated:

I usually say that if there’s no indication for it, then you have to explain to the patient why you have that view. And then, I must say, that really many, if you just take the time to explain the reason for your decision, fully accept it. (GP 3)

#### Tools

Taking time to explain the basis of medical decisions was an important strategy for GPs, not only when assessing the risk for treatment failure, but in all consultations. This would strengthen trust and improve the relationship, which GPs viewed as a core task. An older, experienced GP underlined the importance of this:

And it makes the explanation really important, because if the patient feels he/she does not get the correct treatment because the doctor doesn’t take the illness seriously, then it’s no good right? […] So you must ensure yourself that you have justifiable grounds, but you must also do it to show the patient that you do it on justifiable grounds. (GP 16)

Efforts to maintain a good doctor–patient relationship would commonly involve thorough anamnesis or clinical testing, even when doctors at an early stage were certain of the diagnosis and subsequent management—this to ensure the patients that their worries and reasons for consulting were taken seriously. C-reactive protein (CRP) is commonly used in Nordic primary care as a tool to assess the expected benefit from antibacterial treatment or to monitoring recovery. CRP was mentioned as a helpful diagnostic step but also as an argument in negotiating treatment in RTIs. A younger GP used CRP for this purpose regularly:

I also think you use CRP when there are many viral infections, where you don’t start treatment. In addition, it simply has a pedagogical effect when you say… I just say it beforehand to people: “You know what, if you have a normal infection count, or close to normal, then nothing should happen”. (GP 5)

Other combined clinical and communicative tools GPs used in the clinic were safety netting and wait-and-see prescriptions.

### The patients’ experience according to GPs

In contrast to what GPs themselves understood by treatment failure, they argued that patients probably had a different view. For instance, lack of recovery within a couple of days despite taking antibiotics (i.e. in cases of viral infection) may appear as treatment failure to patients. Patients might also wrongly assume ATF during a lengthy recovery time especially after acute lower RTIs. An experienced doctor (GP 6) had many such examples:

People come and say: “No, I don’t think it’s working.” And then I auscultate and take a CRP, and then we have a clear decrease, and in reality, I have reached effect on regular penicillin. Moreover, when they hear that they will say: “oh but I have also become better, I just thought I would be *a lot* better now”. (GP 6)

GPs argued the patients’ understanding of ATF was incongruent with the GPs’ own medical definition, and thus patients might weigh its importance differently.

## Discussion

### Main findings

The main outcomes from the analysis of antibiotic treatment failure were:

- GPs regarded antibiotic treatment failure unavoidable and defined it in medical terms, i.e. a bacterial infection where the prescribed antibiotic is not effective.
- GPs applied a range of communication strategies to prepare RTI patients for possible change of treatment, anticipating cases of ATF.
- GPs believed patients regard ATF as a more significant phenomenon than doctors did as patients’ understanding of ATF may differ from the medical definition.

### Antibiotic treatment failure and doctor-patient relationship

The participating GPs considered ATF—using their medical definition—an unavoidable problem in their daily work. Experiences in the presented cases of ATF ranged from disappointment to better care due to increased attention. In their work, however, GPs did not feel directly affected by the possibility of treatment failure. A key tool to avoid conflicts and maintain trust with patients was good communication. This included a thorough explanation for choice of treatment, information of possibly prolonged symptoms, and preparing patients for medication change if needed.

Although most GPs would invest time in communicative efforts to level out discrepancies between doctors’ and patients’ understanding of ATF, the GPs considered ATF an issue of minor significance. This could indicate that good communication and a trustful doctor–patient relationship are natural in their daily work. If so, it is a positive result as studies have suggested a correlation between effective doctor–patient communication and improved patient health outcomes [19].

### GPs’ interpretation of the patients’ views

GPs pointed out that patients’ understanding of ATF probably differed from their own medical interpretation. They argued that patients misunderstand the concept and interpret long-lasting symptoms or antibiotic-treated RTIs caused by viral agents incorrectly as ATF. Consequently, GPs proposed that the patients’ views of the issue would be important, as ATF in patients’ terms would cover more cases and thus occur more often. However, this perceived discrepancy between doctors’ and patients’ interpretations of ‘antibiotic treatment failure’ may be incorrect, as our study only captured the doctors’ point of view, and patients may have another story to tell.

### ATF and antibiotic prescriptions

GPs’ impression was that acute RTI patients often came into consultation with different expectations of treatment possibilities than doctors. Interestingly, it has been shown that doctors can think that patients expect a prescription for antibiotics when they actually do not [20,21]. If doctors sense the patient expects a prescription, they have a higher tendency to prescribe antibiotics incorrectly [22]. An inter-European study found that although GPs acknowledge increasing antibiotic usage as a problem they do not consider themselves as contributors to this trend [23].

The current medical definition of ATF causes inherent problems in verifying ATF occurrence, which may explain why doctors tend to ignore the problem [13]. One might argue that if GPs would pair ATF with overprescribing—in line with how GPs believed patients view this issue—it would support a more targeted antibiotic use, limiting treatment to cases with high likelihood of benefit.

### A question of definition

Few studies have investigated ATF and offered a clear definition of the term. Our aim was not to define ATF but to describe GPs’ experiences and understanding of the term. All GPs in the study knew the term and offered their own proposal for a definition, but suggested that the patients defined it differently. This might indicate a need for a second, separate term for the proposed patients’ version.

GPs considered ATF (in their own medical version as well as the patients’ version they proposed) as unavoidable and thus somewhat expected in their daily work. As one GP put it, ‘it’s fundamental.’ This was primarily due to difficulties in discriminating between viral and bacterial aetiologies in acute RTIs. This diagnostic uncertainty is well known and may partly explain why ATF is not considered a major problem.

### Strengths and limitations

To the best of our knowledge, this is the first qualitative study on antibiotic treatment failure in primary care.

One interview (GP 5 and GP 6) combined both a newly educated GP and a more experienced GP. This seemed to give new opportunities for reflection even though they worked together in the same clinic. However, some attitudes might not have surfaced due to lack of privacy in the interview setting.

The interviews were all conducted during the summer, a season with fewer acute RTIs in Denmark. However, acute RTIs are seen all year around and participants had no problems in recalling a case of ATF.

GPs had to respond to the interview invitation and received no compensation for it, which might have skewed the sample as the participating GPs may have had a particular interest in the subject. Many participants (11/18) also did research. This may have contributed to a sample with more restrictive views on antibiotics prescribing, but the sample might also represent broader reflections on the topic in general.

The Nordic culture of prescribing antibiotics differs from the tradition in other European countries, especially southern Europe. This limits the transferability of our study. The interpretation of ATF is quite likely to vary in other national contexts.

As for all qualitative studies, the beliefs and opinions of GPs in this study should not be considered as a general picture but rather as an exploration of a range of views.

### Future perspectives

The increasing consumption of antibiotics, especially of broad spectrum ones, is alarming and warrants action. Antibiotic treatment failure in general practice is a relatively unexplored topic that can help understand factors associated with increasing antibiotic use. New insights in GPs’ views of their prescribing behaviour in acute RTIs can help identify attitudes and behaviours supporting inappropriate antibiotic use. This study explored one relevant aspect, antibiotic treatment failure. It would also be useful to study patient views of ATF, which may be very different.

## Conclusion

Most GPs regard antibiotic treatment failure in medical terms and consider it an unavoidable occurrence in their daily work. ATF may affect the doctor–patient relationship in both negative and positive ways. Doctors hypothesized that patients might have a different view of antibiotic treatment failure. GPs saw many communicative challenges in acute RTI consultations, but overall they thought they were restrictive when prescribing antibiotics and competent in choosing the right treatment. Applying relevant communication strategies in the situation could improve consultations for RTIs; they should especially address perceived differences in understanding and expectations.
